# Effect of dietary supplementation of xylanase alone or combination of xylanase and β-glucanase on growth performance, meat quality, intestinal measurements, and nutrient utilization in broiler chickens

**DOI:** 10.5713/ab.24.0430

**Published:** 2024-10-24

**Authors:** Deok Yun Kim, Kang Hyeon Kim, Eun Cheol Lee, Ju Kyoung Oh, Min Ah Park, Dong Yong Kil

**Affiliations:** 1 Department of Animal Science and Technology, Chung-Ang University, Anseong 17546, Korea; 2 Application Center, CJ Blossom Park, Suwon 16495, Korea

**Keywords:** β-Glucanase, Broiler Chicken, Digesta Viscosity, Growth Performance, Non-starch Polysaccharide, Xylanase

## Abstract

**Objective:**

The current study aimed to investigate the effect of dietary supplementation of xylanase alone or combination of xylanase and β-glucanase in high non-starch polysaccharides (NSP) diets with low energy on growth performance, meat quality, intestinal measurements, stress responses, and energy and nutrient utilization in broiler chickens.

**Methods:**

A total of four hundred 8-d-old Ross 308 broiler chickens were randomly allotted to 1 of 4 treatment groups with 10 replicates. A positive control (PC) diet was formulated with adequate energy and nutrients, whereas a negative control (NC) diet had 100 kcal/kg less nitrogen-corrected apparent metabolizable energy than the PC diet with increasing inclusion of high NSP ingredients. Two additional diets were produced by supplementing 0.1% xylanase alone or 0.1% xylanase and β-glucanase mixture in the NC diet. Experiments lasted for 27 d.

**Results:**

Birds fed PC diets had less (p<0.05) feed conversion ratio (FCR) than those fed NC diets. Birds fed NC diets supplemented with xylanase alone or combination of xylanase and β-glucanase had less (p<0.05) FCR than those fed NC diets. Dietary supplementation of xylanase alone in NC diets exhibited the highest (p<0.05) breast meat pH among dietary treatments. Birds fed PC diets or NC diets supplemented with xylanase and β-glucanase combination exhibited greater (p<0.05) villus height:crypt depth ratio than those fed NC diets. Dietary supplementation of xylanase alone and combination of xylanase and β-glucanase in NC diets decreased (p<0.05) ileal digesta viscosity and increased (p<0.05) xylo-oligosaccharide concentrations in the gastrointestinal tract (GIT) compared with NC diets without affecting energy and nutrient utilization in NC diets.

**Conclusion:**

Dietary supplementation of xylanase in high NSP diets with low energy concentrations improves growth performance by decreasing digesta viscosity and increasing concentrations of xylo-oligosaccharides in the GIT of broiler chickens. However, little additional benefits of β-glucanase supplementation in combination with xylanase are identified for broiler chickens.

## INTRODUCTION

Poultry feeds are primarily composed of plant ingredients rich in non-starch polysaccharides (NSP) that cannot be efficiently utilized by poultry [1]. Among various types of NSP, soluble NSP such as arabinoxylan and β-glucan is well-known to increase digesta viscosity in the gastrointestinal tract (GIT) of poultry, leading to an impairment in intestinal health and function [2,3]. These antinutritional effects of soluble NSP frequently contribute to the reduction in dietary energy and nutrient utilization, thereby decreasing productive performance in poultry [2–4]. In the recent year, increasing feed costs facilitate the use of alternative ingredients such as grain by-products including distillers dried grains with solubles, wheat bran, and rice bran in poultry feeds; however, most of alternative ingredients contain high amounts of soluble NSP, which limits their utilization in poultry feeds [1,2]. As a potential solution, dietary supplementation of NSP-degrading enzymes (NSPase) including xylanase, β-glucanase, and β-mannanase is widely practiced in poultry feeds containing high amounts of soluble NSP because of their ability to mitigate the antinutritional effect of soluble NSP [3–5].

Dietary xylanase is the most common NSPase used in poultry feeds because very high amounts of arabinoxylan as a target soluble NSP of xylanase are present in most feed ingredients [5–7]. There have been mounting evidences that dietary supplementation of xylanase improves productive performance, intestinal health, and energy and nutrient utilization in poultry [4,5,8]. Moreover, previous studies have reported that improved energy and nutrient utilization by dietary xylanase with a combination of other NSPase may save energy and nutrients in diets containing low energy and nutrient concentrations for broiler chickens [9,10].

Considering the nature of high variations in types and amounts of soluble NSP in numerous feed ingredients, the application of the mixture of several NSPases is recently increased in poultry feeds. The reason for this practice is mainly due to the anticipation that the concurrent degradation of various types of soluble NSP would be more effective in mitigating their antinutritional effects than the degradation of single target NSP [9,11,12]. β-Glucanase, which can break down viscous β-glucan in the GIT of poultry [3], is frequently considered the potential choice of NSPase as the combined supplementation of xylanase in poultry feeds [3,11]. Dietary supplementation of xylanase and β-glucanase combination have been reported to improve growth performance and nutrient utilization in broiler chickens [12–14]. However, few studies comparing the effects of dietary supplementation of xylanase alone with those of xylanase and β-glucanase combination in high NSP diets with low energy concentrations have been conducted in broiler chickens.

Therefore, the objective of the current study was to investigate the effect of dietary supplementation of xylanase alone or combination of xylanase and β-glucanase in high NSP diets with low energy concentrations on growth performance, meat quality, intestinal measurements, stress responses, and energy and nutrient utilization in broiler chickens.

## MATERIALS AND METHODS

### Animal ethics statement

All experimental procedures were reviewed and approved by the Institutional Animal Care and the Use Committee at Chung-Ang University (approval No. 202301020009).

### Experiment 1: Growth trial

#### Animals, experimental design, and diets

A total of four hundred 8-d-old Ross 308 broiler chicks (initial body weight [BW]±standard deviation = 191±5.3 g) were allotted to 1 of 4 dietary treatments with 10 replicates in a completely randomized design. Each replicate had 5 male and 5 female birds. All birds were raised in battery cages (76.0×78.0×45.0 cm, width×length×height). A two-phase feeding program was implemented with a grower diet from 8 to 21 d and a finisher diet from 22 to 35 d. Within each phase, a positive control (PC) diet was formulated to meet or exceed the nutrient and energy concentrations recommended in the Ross 308 broiler guideline [15], whereas a negative control (NC) diet was prepared to contain less nitrogen-corrected apparent metabolizable energy (AME_n_) by 100 kcal/kg than the PC diet with increasing inclusion of high NSP ingredients such as wheat, whole rice bran, and defatted rice bran (Table 1). However, the calculated concentrations of crude protein, digestible essential amino acids, total Ca, and available P were equalized between PC and NC diets for both growing and finishing period. Two additional diets were prepared by supplementing 0.1% xylanase (4,000,000 U/kg) or 0.1% xylanase and β-glucanase mixture (4,000,000 U/kg xylanase and 2,000,000 U/kg β-glucanase) to the NC diet in replace of celite. Both enzymes were provided by CJ Bio (Seoul, Korea). All diets were prepared in a mash form.

The experimental diets and water were provided on an *ad libitum* basis for 27 d of feeding trials from 8 to 35 d of age. The room temperature was maintained at 30°C during the first week and then gradually decreased to 20°C at the conclusion of the experiment, following the recommendation outlined in the Ross 308 broiler guideline [15]. The average relative humidity was 49%±13.5% during the experiment. The experiment was conducted under a 23-h lighting scheme. The BW gain (BWG) and feed intake (FI) were recorded at the conclusion of the experiment. Mortality was documented daily. Feed conversion ratio (FCR) was calculated by dividing FI by BWG after adjusting for mortality [16].

#### Sample collection and analysis

At the end of the experiment, 1 male broiler chicken with a BW closest to the average BW per replicate was euthanized using CO_2_ asphyxiation. This bird was used for the analysis of meat quality, jejunal morphology, digesta viscosity, intestinal concentrations of xylo-oligosaccharides (XOS), and blood heterophil to lymphocyte ratio (H:L ratio). The blood sample was immediately collected from each bird via a heat puncture into a 10-mL EDTA tube (Becton and Dickinson company, Diagnostics, Wokingham, UK). Blood H:L ratio was analyzed by the method of Lentfer et al [17] with a minor modification. The detailed procedure was reported in our previous experiment [18]. The breast meat was collected for the analysis of pH, meat colors, and water holding capacity (WHC), which were analyzed by the previous methods [16].

Jejunal fragments were collected to analyze the jejunal morphology. Approximately 1 cm section of the jejunum was flushed and fixed with 10% buffered formalin. Fixed samples were embedded, sectioned, and stained with hematoxylin and eosin stain. The villus height (VH), crypt depth (CD), villus width (VW), and VH:CD ratio were measured following the method of Wiersema et al [19] with a minor modification. Twenty measurements were taken per jejunal sample, the average value from these measurements was calculated to represent each measurement. This analysis was performed at the BT research facility center, Chung-Ang University.

Digesta samples were collected from both the jejunum and ileum for the analysis of digesta viscosity. The digesta samples were centrifuged at 2,800×*g* (5810R; Eppendorf, Hamburg, Germany) at 20°C for 15 min. Supernatants were then transferred to 15 mL conical tubes. The viscosity measurement was conducted with 500 μL of supernatant using a viscometer (DV2T; AMETEK, Brookfield, MA, USA) set at 20 and 50 rpm under 39°C for 20 s [7]. In addition, total XOS concentrations in the jejunal and ileal digesta were also determined. Briefly, 1 mL of 5 times diluted digesta was mixed with 1.33 mL methanol and 2.66 mL chloroform. The supernatant obtained after centrifugation at 1,580×g and 20°C for 3 min was evaporated using a centrifugal vacuum concentrator (HyperVac-Lite; Hanil Scientific Inc., Gimpo, Korea). The dried pellet was resuspended in 0.5 mL of 50% methanol and filtered with a C8 cartridge (Sep-Pak C8 Vac RC; Waters, Milford, MA, USA) followed by a 0.22 μm syringe filter. Approximately 2 μL of filtered samples were used to separate and quantify each XOS concentration using a liquid chromatography-mass specterometry. Total XOS concentrations were then calculated by summing individual XOS from xylobiose to xylohexaose. The detailed procedure for the anlaysis of digesta viscosity and total XOS concentrations was reported previously [7].

### Experiment 2: *In vitro* digestion study to predict enzyme efficacy

The artificial GIT system specifically designed for broiler chickens in the CJ BIO Animal Nutrition and Health (ANH) Application Platform [7] was employed to assess the efficacy of dietary supplementation of xylanase alone or combination of xylanase and β-glucanase in *in vitro* digestion trial. Briefly, 2 treatment diets (i.e., PC and NC diets) used in the growing period were ground to a fine particle (i.e., less than 1 mm) and 27 g of each diet were resuspended in 500 mL of phosphate buffer at pH 5.0. Afterwards, either xylanase alone or combination of xylanase and β-glucanase was supplemented to NC diets at the same activity levels as those used in the growth trial, whereas calcium carbonate was supplemented to both PC and NC diets at the equivalent amount of supplemental enzymes. The digestion process was conducted in triplicate using *in vitro* digestion model at 700 rpm under 40°C. *In vitro* digesta samples were collected from each digestion jar after 270 min of digestion. Total XOS concentrations in *in vitro* digesta were measured and calculated as determined in the jejunal and ileal digesta from the growth trial. The detailed procedure was described in the previous study [7].

### Experiment 3: Metabolism trial

#### Experimental design, sample collection, and analysis

A total of forty 40-d-old male chickens were selected at the end of the growth trial and assigned to 1 of 4 dietary treatments with 10 replicates. Each replicate had 1 male bird. All birds had similar BW at the start of the metabolism trial and were continuously fed the same treatment diets used in finishing period of the growth trial. The detailed procedure in the metabolism trial was outlined in our previous study [20].

Excreta were collected daily and immediately stored at −20°C. The excreta samples were dried in a forced-air drying oven at 60°C for 48 h and finely ground for further analyses. Both diets and excreta samples were analyzed for dry matter (method 934.01;), nitrogen (N; method 990.03) according to the methods described in AOAC [21], and for gross energy (GE) using bomb calorimetry (Model 6400; Parr Instruments Co., Moline, IL, USA). The concentrations of total Ca and P in both diets and excreta were analyzed using an inductively coupled plasma spectrometer (Optima 5300 DV; Perkin Elmer Inc., Shelton, CT, USA), following the method outlined by AOAC (Method 935.13; [21]) with a minor modification [22].

Apparent total tract retention (ATTR) of GE, N, Ca, and P in treatment diets were calculated based on the previous method [23]. The values for apparent metabolizable energy (AME) and AME_n_ were also calculated with determined values for the ATTR of GE and N [20,24].

### Statistical analysis

All data were analyzed in a completely randomized design using PROC MIXED procedure of SAS (SAS Institute., Cary, NC, USA). Each replicate was considered an experimental unit for all analyses. All data were checked to find the presence of outlier data using the UNIVARIATE procedure of SAS. The LSMEANS procedure was used to calculate treatment means and the PDIFF option of SAS was used to separate the means if the difference was significant. Significance level for statistical tests was set at p<0.05.

## RESULTS

### Growth performance and breast meat quality

Birds fed PC diets had less (p<0.05) FCR than those fed NC diets (Table 2). Birds fed NC diets supplemented with xylanase alone or combination of xylanase and β-glucanase had also less (p<0.05) FCR than those fed NC diets. No differences in FCR were observed between birds fed PC diets and those fed NC diets supplemented with xylanase alone or combination of xylanase and β-glucanase. However, dietary treatments had no effects on BW, BWG, and FI in broiler chickens.

No differences in all breast meat qualities were observed between birds fed PC diets and those fed NC diets, except b* values being less (p<0.05) in NC treatment than in PC treatment (Table 3). Dietary supplementation of xylanase alone or combination of xylanase and β-glucanase in NC diets did not affect pH and WHC in the breast meat as compared to NC diets. However, birds fed NC diets supplemented with xylanase alone exhibited the highest (p<0.05) pH at 24-h postmortem among dietary treatments. Interestingly, dietary supplementation of xylanase alone and combination of xylanase and β-glucanase in NC diets decreased (p<0.05) a* values in the breast meat as compared to NC diets with no impact on L* and b* values.

### Jejunal morphology

No differences in VH and VW were observed among dietary treatments (Table 4). However, birds fed PC diets had the least (p<0.05) CD, whereas those fed NC diets had the greatest (p<0.05) CD among dietary treatments. Likewise, birds fed PC diets or NC diets supplemented with xylanase and β-glucanase combination had greater (p<0.05) VH:CD ratio than those fed NC diets or NC diets supplemented with xylanase alone.

### Digesta viscosity

**Figure 1:**
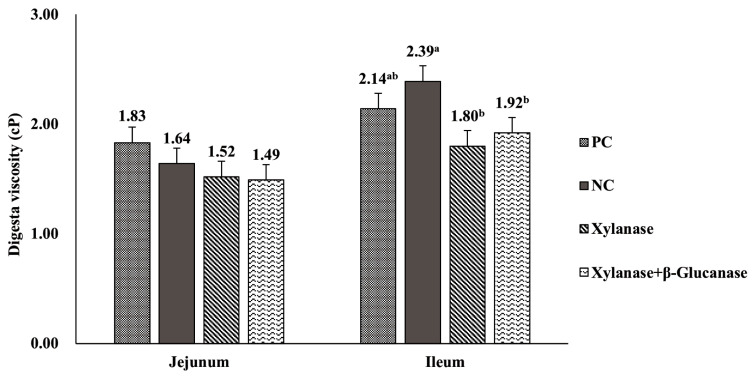
Effect of dietary supplementation of xylanase alone or combination of xylanase and β-glucanase on digesta viscosity in the jejunum and ileum of broiler chickens. Data are presented with least squares means and standard error of the means (10 observations per treatment). PC, positive control (basal diets with adequate energy and nutrients); NC, negative control (high-NSP diets and 100 kcal/kg AME_n_ less than PC diet); Xylanase, NC+0.1% xylanase (4,000,000 units/kg); Xylanase+β-Glucanase, NC+0.1% xylanase-glucanase complex (4,000,000 units/kg xylanase and 2,000,000 units/kg β-glucanase). AME_n_, nitrogen-corrected apparent metabolizable energy. ^a,b^ Means within a variable with no common superscript differ significantly (p<0.05).

Dietary treatments did not influence jejunal digesta viscosity (Figure 1). No differences in ileal digesta viscosity were observed between birds fed PC diets and those fed NC diets. However, dietary supplementation of xylanase alone and combination of xylanase and β-glucanase in NC diets decreased (p<0.05) ileal digesta viscosity as compared to NC diets. No differences in ileal digesta viscosity were found between dietary supplementation of xylanase alone and combination of xylanase and β-glucanase in NC diets

### *In vitro* and *in vivo* analysis of xylo-oligosaccharides

In *in vitro* digestion trial, total XOS concentrations in digesta did not differ between PC and NC treatments (Table 5). Supplementation of xylanase alone and combination of xylanase and β-glucanase in NC diets increased (p<0.05) total XOS concentrations in digesta as compared to those obtained from NC treatments. However, no differences in total XOS concentrations were observed between supplementation of xylanase alone and combination of xylanase and β-glucanase.

In the growth trial, feeding diets supplemented with xylanase alone and combination of xylanase and β-glucanase to broiler chickens increased (p<0.05) total XOS concentrations in the jejunal and ileal digesta compared with feeding PC or NC diets. Total XOS concentrations were greater (p<0.05) for dietary supplementation of xylanase and β-glucanase combination than for supplementation of xylanase alone, which were identified in both jejunal and ileal digesta of broiler chickens.

### Blood heterophil to lymphocyte ratio

**Figure 2:**
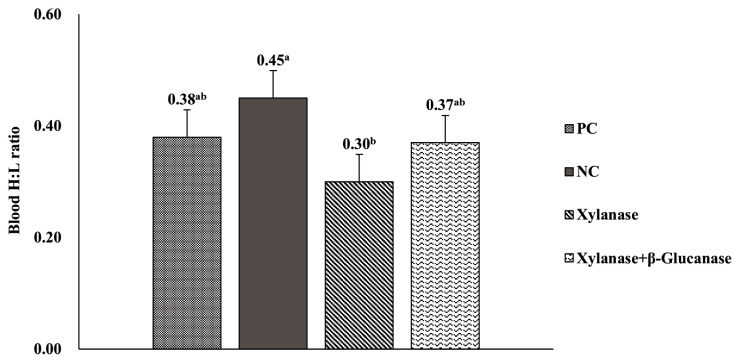
Effect of dietary supplementation of xylanase alone or combination of xylanase and β-glucanase on blood heterophil to lymphocyte ratio (H:L ratio) in broiler chickens. Data are presented with least squares means and standard error of the means (10 observations per treatment). PC, positive control (basal diets with adequate energy and nutrients); NC, negative control (high-NSP diets and 100 kcal/kg AME_n_ less than PC diet); Xylanase, NC+0.1% xylanase (4,000,000 units/kg); Xylanase+β-Glucanase, NC+0.1% xylanase-glucanase complex (4,000,000 units/kg xylanase and 2,000,000 units/kg β-glucanase). AME_n_, nitrogen-corrected apparent metabolizable energy. ^a,b^ Means within a variable with no common superscript differ significantly (p<0.05).

Blood H:L ratio did not differ between birds fed PC diets and those fed NC diets (Figure 2). However, dietary supplementation of xylanase alone in NC diets decreased (p<0.05) blood H:L ratio compared with NC diets. Birds fed PC diets or NC diets supplemented with xylanase and β-glucanase combination had no different blood H:L ratio compared with those fed NC diets supplemented with xylanase alone.

### Energy and nutrient utilization in treatment diets

The NC diets exhibited less (p<0.05) ATTR of GE, AME, and AME_n_ than PC diets (Table 6). However, dietary supplementation of xylanase alone and combination of xylanase and β-glucanase in NC diets had no impact on the ATTR of GE, AME, and AME_n_ in NC diets. Similarly, no differences in the ATTR of N, Ca, and P were observed among treatment diets.

## DISCUSSION

High concentrations of NSP, in particular for soluble NSP such as arabinoxylan, β-glucan, and β-mannan in diets are documented to depress productive performance in broiler chickens. This result is primarily associated with increasing digesta viscosity, which can exert adverse effects on intestinal development, microbial population, and nutrient utilization [1–3]. Consistent with the findings of previous studies, the current study also revealed that NC diets with increasing inclusion of high NSP ingredients such as wheat and rice bran resulted in decreased broiler performance with impaired intestinal morphology and nutrient utilization, despite little impacts on digesta viscosity measured in the jejunum and ileum. However, it is important to note that NC diets used in this study were specifically designed to contain less amount of AME_n_ by 100 kcal/kg than PC diets. Therefore, the reduction in broiler performance by feeding NC diets as observed in the current study was likely caused by both high NSP and low energy concentrations in diets.

Dietary xylanase is the most commonly used in poultry diets because the arabinoxylan as a target NSP of xylanase is present at high amounts in corn and wheat that are conventional energy ingredients in poultry diets [4,6–8]. Previous studies have reported that dietary supplementation of xylanase improved productive performance in broiler chickens [4,6,8]. This beneficial effect of dietary xylanase has been primarily attributed to its ability to decrease digesta viscosity by breaking down viscous arabinoxylan in the GIT of broiler chickens [4,6,8]. Furthermore, decreased digesta viscosity in the GIT of broiler chickens is reported to enhance intestinal functions and prevent undesirable microbial fermentation, thereby contributing to the improvement in intestinal morphological structure and utilization of energy and nutrients in diets [5,6]. Moreover, it has also been demonstrated that improved intestinal health by dietary supplementation of xylanase is linked to potential prebiotic effects of xylanase because dietary xylanase can enhance prebiotic XOS concentrations in the GIT of broiler chickens [4–6]. Therefore, we hypothesized that dietary supplementation of xylanase in high NSP diets with low energy concentrations may both compensate for reduced energy concentrations in diets and mitigate adverse effects of soluble NSP by enhancing intestinal health and function with improving energy and nutrient utilization in broiler chickens.

In the present study, comparable productive performance was observed in broiler chickens fed NC diets supplemented with xylanase alone to those fed PC diets. This finding suggests that dietary supplementation of 0.1% xylanase (equivalent to 4,000 U/kg in diets) used in this study may save approximately 100 kcal/kg AME_n_ in high NSP diets for broiler chickens. This positive effect was likely associated with our observations of decreased digesta viscosity, increased concentrations of prebiotic XOS in the jejunal and ileal digesta, and increased VH:CD ratio, although significance for VH:CD ratio was not detected. Interestingly, the relatively small and non-significant increase was observed in measured AME_n_ values by dietary supplementation of xylanase in NC diets (3,072 vs 3,034 kcal/kg). Therefore, it can be inferred that improved productive performance in broiler chickens by dietary supplementation of xylanase in high NSP diets with low energy concentrations may not be solely attributed to improved energy and nutrient utilization. Instead, it may also be related to other physiological benefits, including an improvement in intestinal health and function, possibly by increasing prebiotic XOS contents in the GIT [4–6], as well as a decrease in stress responses (i.e., blood H:L ratio) as observed in the current study.

Given the considerable variability in types and amounts of soluble NSP among diverse feed ingredients, the use of NSPase mixtures is increasingly applied in the poultry industry. This approach is based on the expectation of possible cooperative actions among individual NSPases in the mixtures to simultaneously degrade various types of soluble NSP. One potential NSPase suitable for the combined supplementation with xylanase in poultry diets is β-glucanase. This NSPase can specifically target and degrade viscous β-glucan in the GIT of poultry, thereby mitigating its antinutritional effects on productive performance and health in poultry [3,11]. Previous studies have reported that dietary supplementation of xylanase and β-glucanase combination improved growth performance and nutrient utilization in broiler chickens possibly by decreasing digesta viscosity and favoring microbial populations [12–14]. Moreover, these studies have revealed that the positive effects were more pronounced in wheat-based diets compared with corn-based diets [12–14]. However, there remains a lack of experiments comparing the effect of dietary supplementation of xylanase alone with those of xylanase and β-glucanase combination in broiler chickens. One previous study reported that combined use of xylanase and β-glucanase in corn and soybean meal-based diets exerted no fully additive effects on broiler performance as compared to the effect of individual use of xylanase and β-glucanase although some additivities for improvements in amino acid and energy utilization were identified [25]. Similarly, we also found no significant differences in growth performance, digesta viscosity, blood H:L ratio, and utilization of energy and nutrients between dietary supplementation of xylanase alone and the combination of xylanase and β-glucanase, despite the inclusion of increasing amounts of high NSP ingredients such as wheat and rice bran in treatment diets. The lack of additional benefits of β-glucanase in combination of xylanase in broiler diets may be associated with the relatively low concentrations of β-glucan in wheat and rice bran used in the experimental diets [1,26]. Moreover, it has been postulated that individual NSPase in the mixture of NSPases may have a limitation to exert their maximal impacts, possibly due to competition for similar types of NSP substrates and overlap in nutritional benefits of individual NSPases [25]. In the current study, however, dietary supplementation of both xylanase and β-glucanase resulted in increased total XOS concentrations in the GIT of broiler chickens to a greater extent than dietary supplementation of xylanase alone, indicating that dietary β-glucanase may enhance XOS profiles in the GIT of broiler chickens with a possible synergistic action of dietary xylanase. Thus, it is likley that combined use of xylanase and β-glucanase in diets may boost the prebiotic effect of xylanase alone in broiler chickens and this benefit may expand if broiler diets are formulated with increasing inclusion levels of high NSP ingredients such as wheat bran, rice bran, copra meal, and palm kernel meal, which are largely used in many Asian countries. Neverthless, the reason for the lack of additional benefits from combined use of β-glucanase with xylanase in broiler diets as observed in this study is not clearly known. Further research regarding the comparative effect of dietary xylanase and β-glucanase combination with individual xylanase and β-glucanase on productive performance and intestinal health in poultry is required.

In both *in vitro* digestion and growth trials, appreciable amounts of XOS were detected in the digesta from PC and NC treatments despite no supplementation of enzymes in PC and NC diets. This observation is consistent with the previous study reporting that endogenous xylanase present in wheat sources as well as in the GIT of broiler chickens could partially hydrolyze dietary arabinoxylan complex [27]. The supplementation of xylanase alone increased total XOS concentrations in *in vitro* digesta, confirming our results for increasing total XOS concentrations measured directly in both jejunal and ileal digesta of broiler chickens. This result suggested that the artificial GIT system used in *in vitro* digestion study [7] may serve as a useful tool to predict the efficacy of exogenous digestive enzymes in broiler chickens. However, dietary supplementation of xylanase and β-glucanase combination in NC diets increased total XOS concentrations in both jejunal and ileal digesta of broiler chickens to a greater extent than that of xylanase alone, whereas this difference was not fully identified in *in vitro* digestion system. The reason for this variation may be related to the relatively low ability of *in vitro* digestion systems to completely realize the digestive physiology in poultry [28,29]. Despite these limitations, the use of *in vitro* digestive system may still be valuable for conducting preliminary assessments of dietary treatments in animal nutrition studies.

Changes in nutrient availability and utilization may influence biochemical process in the muscle tissue, potentially altering the meat quality of broiler chickens [30]. In the current study, significant changes in breast meat pH were observed at 24-h postmortem with dietary supplementation of xylanase alone, leading to the greatest pH at 24-h postmortem among dietary treatments. In addition, a* values in meat color were decreased by dietary supplementation of xylanase alone or combination of xylanase and β-glucanase in NC diets without affecting L* and b* values. The reason for these observations is unclear because the data pertaining to the effect of alterations in specific nutrients and their utilization by dietary enzyme supplementation on meat pH and color in broiler chickens are lacking. However, reduction in b* values by feeding NC diets or NC diets supplemented with enzymes may be linked to decreased inclusion levels of corn and corn gluten meal, which contain high amounts of yellow-colored xanthophyll, in those treatment diets because b* (yellowness) values in poultry meat are highly dependent on the amount of dietary xanthophyll intake [31]. However, it appears that all values for meat quality measured in this study fell within representative ranges determined in the conventional broiler breast meat.

Blood H:L ratio is widely recognized as a stress biomarker in poultry with its elevated levels indicating increased stress responses due to various environmental and physiological stressors [32]. Nutritional deficiency is also considered a critical stressor in animals. Previous studies have demonstrated that decreasing energy or nutrient concentrations in diets as compared to their required levels may increase stress responses in broiler chickens [33–35]. This finding was further confirmed by our observation that broiler chickens fed NC diets had increased blood H:L ratio than those fed PC diets, albeit not reaching a statistical significance. Moreover, birds fed diets supplemented with xylanase alone or combination of xylanase and β-glucanase exhibited decreased blood H:L ratio, which was similar to those fed PC diets, providing an indirect evidence of improved energy and nutrient utilization by supplementing these enzymes in high NSP diets with low energy concentrations. In the metabolism trial, however, dietary supplementation of xylanase alone or combination of xylanase and β-glucanase in NC diets did not affect energy and nutrient utilization in NC diets, despite slight improvements in AME and AME_n_ values being observed. Therefore, it is speculated that increasing prebiotic XOS concentrations in the GIT of broiler chickens by dietary supplementation of xylanase alone or combination of xylanase and β-glucanase may contribute to decreased stress responses. It has been reported that dietary supplementation of prebiotic oligosaccharides can decrease stress responses by promoting microbial ecosystems and enhancing immune systems in the GIT of broiler chickens [4–6].

## CONCLUSION

Dietary supplementation of xylanase alone in high NSP diets with low energy concentrations improved growth performance in broiler chickens. This improvement was likely achieved by decreasing digesta viscosity, increasing concentrations of total XOS in the GIT, and decreasing stress responses. However, the combined supplementation of β-glucanase with xylanase in high NSP diets with low energy concentrations had little additional benefits for broiler chickens.

## Figures and Tables

**Table 1:** Composition and nutrient concentrations of the experimental diets

Items	Grower phase (8 to 21 d)		Finisher phase (22 to 35 d)	
	
	PC^1)^	NC^1)^	PC^1)^	NC^1)^
Ingredients (%)				
Corn grains	45.151	29.930	49.812	29.544
Soybean meal, 45% CP	25.650	24.220	14.860	20.610
Corn gluten meal	5.880	5.100	9.610	4.730
Wheat	10.000	25.000	10.000	25.000
Whole rice bran	1.500	3.500	2.500	5.000
Defatted rice bran	1.500	3.500	2.500	5.000
Soybean oil	4.630	3.120	4.900	4.760
MDCP	1.556	1.360	1.434	1.203
Limestone	1.427	1.503	1.392	1.431
L-Lysine HCl (78.5%)	0.369	0.385	0.509	0.387
DL-Methionine (98%)	0.653	0.661	0.600	0.635
L-Threonine (99%)	0.185	0.200	0.191	0.185
L-Tryptophan (98%)	0.017	0.009	0.042	0.012
L-Valine (98.5%)	0.063	0.075	0.076	0.073
L-Isoleucine (99%)	0.054	0.066	0.088	0.073
L-Arginine (99%)	0.165	0.171	0.286	0.157
Celite	0.200	0.200	0.200	0.200
NaCl	0.200	0.200	0.200	0.200
Choline (50%)	0.100	0.100	0.100	0.100
NaHCO_3_	0.200	0.200	0.200	0.200
Coccidiostats	0.100	0.100	0.100	0.100
Antioxidant	0.100	0.100	0.100	0.100
Vitamin premix^2)^	0.150	0.150	0.150	0.150
Mineral premix^3)^	0.150	0.150	0.150	0.150
Total	100.000	100.000	100.000	100.000
Calculated energy and nutrient content^4)^				
AME_n_ (kcal/kg)	3,100	3,000	3,200	3,100
Crude protein (%)	21.50	21.50	20.00	20.00
Crude fat (%)	7.12	5.69	7.71	7.60
Crude fiber (%)	2.61	2.90	2.45	2.98
Digestible Lys (%)	1.15	1.15	1.08	1.08
Digestible Met (%)	0.95	0.95	0.90	0.90
Digestible Thr (%)	0.77	0.77	0.71	0.71
Digestible Trp (%)	0.18	0.18	0.17	0.17
Digestible Ile (%)	0.78	0.78	0.73	0.73
Digestible Arg (%)	1.23	1.23	1.13	1.13
Digestible Val (%)	0.87	0.87	0.81	0.81
Digestible Gly+Ser (%)	1.48	1.47	1.31	1.36
Total calcium (%)	0.87	0.87	0.81	0.81
Available phosphorus (%)	0.44	0.44	0.41	0.41
Nonstarch polysaccharides (NSP)^5)^				
Soluble NSP (g/kg)	11.6	14.1	8.5	12.8
Insoluble NSP (g/kg)	69.2	72.2	66.5	70.2
Total NSP (g/kg)	80.8	86.3	74.9	83.0

CP, crude protein; MDCP, mono-dicalcium phosphate; AME_n_, nitrogen-corrected apparent metabolizable energy; NSP, non-starch polysaccharides

1) PC, positive control (basal diets with adequate energy and nutrients); NC, negative control (high-NSP diets and 100 kcal/kg AME_n_ less than PC diet).

2) Provided per kg of the complete diet: vitamin A, 12,000 IU (retinyl acetate); vitamin D_3_, 4,000 IU; vitamin E, 80.0 mg; vitamin K_3_, 4.0 mg (menadione dimethylpyrimidinol); vitamin B_1_, 4.0 mg; vitamin B_2_, 10.0 mg; vitamin B_6_, 6.0 mg; vitamin B_12_, 20.0 μg; folic acid, 2.0 mg; biotin, 200 μg; niacin, 60 mg.

3) Provided per kg of the complete diet: iron, 60 mg (FeSO_4_); zinc, 100 mg (ZnSO_4_); manganese, 120 mg (MnO); copper, 16 mg (CuSO_4_); cobalt, 1,000 μg (CoSO_4_); selenium, 300 μg (Na_2_SeO_3_); iodine, 1.25 mg (Ca[IO_3_]_2_).

4) Calculated values from CVB [36].

5) Analyzed values [37].

**Table 2:** Effect of dietary supplementation of xylanase alone or combination of xylanase and β-glucanase on growth performance in broiler chickens (8 to 35 d)

Treatments^1)^	Growth performance			

	BW (g)	BWG (g)	FI (g)	FCR (g/g)
PC	2,085	1,894	2,900	1.53^b^
NC	2,008	1,818	2,876	1.59^a^
Xylanase	2,030	1,840	2,807	1.53^b^
Xylanase+β-glucanase	2,067	1,877	2,871	1.53^b^
SEM	230.7	235.2	335.7	0.021
p-value	0.303	0.297	0.303	0.032

Data are least squares means of 10 observations per treatment.

BW, body weight; BWG, body weight gain; FI, feed intake; FCR, feed conversion ratio; SEM, standard error of means; AME_n_, nitrogen-corrected apparent metabolizable energy.

1) PC, positive control (basal diets with adequate energy and nutrients); NC, negative control (high-NSP diets and 100 kcal/kg AME_n_ less than PC diet); Xylanase, NC+0.1% xylanase (4,000,000 units/kg); Xylanase+β-Glucanase, NC+0.1% xylanase-glucanase complex (4,000,000 units/kg xylanase and 2,000,000 units/kg β-glucanase).

a,b Means within a variable with no common superscript differ significantly (p<0.05).

**Table 3:** Effect of dietary supplementation of xylanase alone or combination of xylanase and β-glucanase on breast meat quality in broiler chickens

Treatments^1)^	Breast meat quality					

	pH (1 h)	pH (24 h)	WHC (%)	Meat color		

				L*	a*	b*
PC	5.91	5.65^b^	69.53	48.1	4.4^ab^	17.0^a^
NC	6.00	5.69^ab^	71.43	48.3	5.3^a^	15.2^b^
Xylanase	5.96	5.75^a^	70.29	47.7	3.2^b^	14.0^b^
Xylanase+β-glucanase	6.00	5.65^b^	68.14	48.4	3.4^b^	14.5^b^
SEM	0.138	0.133	3.764	2.09	0.56	1.50
p-value	0.539	0.039	0.452	0.963	0.040	0.001

Data are least squares means of 10 observations per treatment.

WHC, water holding capacity; L*, lightness; a*, redness; b*, yellowness; SEM, standard error of means; AME_n_, nitrogen-corrected apparent metabolizable energy.

1) PC, positive control (basal diets with adequate energy and nutrients); NC, negative control (high-NSP diets and 100 kcal/kg AME_n_ less than PC diet); Xylanase, NC+0.1% xylanase (4,000,000 units/kg); Xylanase+β-Glucanase, NC+0.1% xylanase-glucanase complex (4,000,000 units/kg xylanase and 2,000,000 units/kg β-glucanase).

a,b Means within a variable with no common superscript differ significantly (p<0.05).

**Table 4:** Effect of dietary supplementation of xylanase alone or combination of xylanase and β-glucanase on the jejunal morphology in broiler chickens

Treatments^1)^	Jejunal morphology			

	VH (μm)	CD (μm)	VW (μm)	VH:CD ratio
PC	1,292	145^c^	147	9.54^a^
NC	1,343	184^a^	159	7.91^b^
Xylanase	1,314	168^ab^	160	8.19^b^
Xylanase+β-glucanase	1,419	157^bc^	161	9.45^a^
SEM	210.6	6.9	12.2	1.552
p-value	0.159	<0.001	0.475	<0.001

Data are least squares means of 10 observations per treatment.

VH, villus height; CD, crypt depth; VW, villus width; VH:CD ratio, villus height to crypt depth ratio; SEM, standard error of means; AME_n_, nitrogen-corrected apparent metabolizable energy.

1) PC, positive control (basal diets with adequate energy and nutrients); NC, negative control (high-NSP diets and 100 kcal/kg AME_n_ less than PC diet); Xylanase, NC+0.1% xylanase (4,000,000 units/kg); Xylanase+β-glucanase, NC+0.1% xylanase-glucanase complex (4,000,000 units/kg xylanase and 2,000,000 units/kg β-glucanase).

a–c Means within a variable with no common superscript differ significantly (p<0.05).

**Table 5:** Effect of dietary supplementation of xylanase alone or combination of xylanase and β-glucanase on total concentrations of xylo-oligosaccharides in *in vitro* digestion and *in vivo* growth trial^1)^

Treatments^2)^	Total concentrations of xylo-oligosaccharides (ppm)		

	*In vitro* digestion	*In vivo* growth trial	

		Jejunum	Ileum
PC	6.1^b^	17.0^c^	19.9^c^
NC	6.2^b^	13.7^c^	7.9^c^
Xylanase	16.7^a^	50.2^b^	81.5^b^
Xylanase+β-glucanase	18.3^a^	104.0^a^	252.4^a^
SEM	1.06	10.83	21.82
p-value	<0.001	<0.001	<0.001

Data are least squares means of 3 and 7 observations per treatment for *in vitro* digestion and *in vivo* growth trial, respectively.

SEM, standard error of means; AME_n_, nitrogen-corrected apparent metabolizable energy.

1) Total concentrations of xylo-oligosaccharides were calculated by summing individual xylo-oligosaccharides from xylobiose (X^2)^ to xylohexaose (X6).

2) PC, positive control (basal diets with adequate energy and nutrients); NC, negative control (high-NSP diets and 100 kcal/kg AME_n_ less than PC diet); Xylanase, NC+0.1% xylanase (4,000,000 units/kg); Xylanase+β-Glucanase, NC+0.1% xylanase-glucanase complex (4,000,000 units/kg xylanase and 2,000,000 units/kg β-glucanase).

a–c Means within a variable with no common superscript differ significantly (p<0.05).

**Table 6:** Effect of dietary supplementation of xylanase alone or combination of xylanase and β-glucanase on apparent total tract retention (ATTR) of nutrients and metabolizable energy (ME) in broiler diets

Treatments^1)^	ATTR (%)				ME value (kcal/kg)	
	
	GE	N	Ca	P	AME	AME^n^
PC	81.97^a^	61.17	47.54	47.32	3,378^a^	3,217^a^
NC	78.62^b^	62.51	51.96	44.87	3,199^b^	3,034^b^
Xylanase	79.50^b^	62.04	48.03	42.58	3,235^b^	3,072^b^
Xylanase+β-glucanase	79.50^b^	62.98	52.27	45.21	3,235^b^	3,069^b^
SEM	0.604	1.038	2.933	2.690	24.7	23.5
p-value	<0.001	0.634	0.128	0.247	<0.001	<0.001

Data are least squares means of 10 observations per treatment.

GE, gross energy; N, nitrogen; Ca, calcium; P, phosphorus; AME, apparent metabolizable energy; AME_n_, nitrogen-corrected apparent metabolizable energy; SEM, standard error of means.

1) PC, positive control (basal diets with adequate energy and nutrients); NC, negative control (high-NSP diets and 100 kcal/kg AME_n_ less than PC diet); Xylanase, NC+0.1% xylanase (4,000,000 units/kg); Xylanase+β-Glucanase, NC+0.1% xylanase-glucanase complex (4,000,000 units/kg xylanase and 2,000,000 units/kg β-glucanase).

a,b Means within a variable with no common superscript differ significantly (p<0.05).

## References

[1] Knudsen KEB (1997). Carbohydrate and lignin contents of plant materials used in animal feeding. Anim Feed Sci Technol.

[2] Choct M (2015). Feed non-starch polysaccharides for monogastric animals: classification and function. Anim Prod Sci.

[3] Bedford Ma, Schulze H (1998). Exogenous enzymes for pigs and poultry. Nutr Res Rev.

[4] Baker JT, Duarte ME, Holanda DM, Kim SW (2021). Friend or foe? impacts of dietary xylans, xylooligosaccharides, and xylanases on intestinal health and growth performance of monogastric animals. Animals.

[5] Bedford MR, Apajalahti JH (2022). The role of feed enzymes in maintaining poultry intestinal health. J Sci Food Agric.

[6] Kiarie E, Romero LF, Nyachoti CM (2013). The role of added feed enzymes in promoting gut health in swine and poultry. Nutr Res Rev.

[7] Kim HY, Moon J, Kim SW (2024). Development and application of a multi-step porcine in vitro system to evaluate feedstuffs and feed additives for their efficacy in nutrient digestion, digesta characteristics, and intestinal immune responses. Anim Nutr.

[8] Kiarie E, Romero LF, Ravindran V (2014). Growth performance, nutrient utilization, and digesta characteristics in broiler chickens fed corn or wheat diets without or with supplemental xylanase. Poult Sci.

[9] Coppedge JR, Oden LA, Ratliff B, Brown B, Ruch F, Lee JT (2012). Evaluation of nonstarch polysaccharide-degrading enzymes in broiler diets varying in nutrient and energy levels as measured by broiler performance and processing parameters. J Appl Poult Res.

[10] Saleh AA, Kirrella AA, Abdo SE (2019). Effects of dietary xylanase and arabinofuranosidase combination on the growth performance, lipid peroxidation, blood constituents, and immune response of broilers fed low-energy diets. Animals.

[11] Slominski BA (2011). Recent advances in research on enzymes for poultry diets. Poult Sci.

[12] Munyaka PM, Nandha NK, Kiarie E, Nyachoti CM, Khafipour E (2016). Impact of combined β-glucanase and xylanase enzymes on growth performance, nutrients utilization and gut microbiota in broiler chickens fed corn or wheat-based diets. Poult Sci.

[13] Mathlouthi N, Mallet S, Saulnier L, Quemener B, Larbier M (2002). Effects of xylanase and β-glucanase addition on performance, nutrient digestibility, and physico-chemical conditions in the small intestine contents and caecal microflora of broiler chickens fed a wheat and barley-based diet. Anim Res.

[14] Kouzounis D, Kers JG, Soares N, Smidt H, Kabel MA, Schols HA (2022). Cereal type and combined xylanase/glucanase supplementation influence the cecal microbiota composition in broilers. J Anim Sci Biotechnol.

[15] Aviagen (2019). Ross 308 broiler: nutrition specifications.

[16] Choi WJ, Kim JH, Han GP, Kwon CH, Kil DY (2021). Effects of dietary hatchery by-products on growth performance, relative organ weight, plasma measurements, immune organ index, meat quality, and tibia characteristics of broiler chickens. Anim Biosci.

[17] Lentfer TL, Pendl H, Gebhardt-Henrich SG, Fröhlich E, Von Borell E (2015). H/L ratio as a measurement of stress in laying hens–methodology and reliability. Br Poult Sci.

[18] Kim DY, Kim JH, Choi WJ, Han GP, Kil DY (2021). Comparative effects of dietary functional nutrients on growth performance, meat quality, immune responses, and stress biomarkers in broiler chickens raised under heat stress conditions. Anim Biosci.

[19] Wiersema ML, Koester LR, Schmitz-Esser S, Koltes DA (2021). Comparison of intestinal permeability, morphology, and ileal microbial communities of commercial hens housed in conventional cages and cage-free housing systems. Poult Sci.

[20] Lee JH, Kwon CH, Won SY, Kim HW, Kil DY (2023). Evaluation of tryptophan biomass as an alternative to conventional crystalline tryptophan in broiler diets. J Appl Poult Res.

[21] AOAC International (2007). Official methods of analysis of AOAC International.

[22] Kim JW, Kim JH, Shin JE, Kil DY (2016). Relative bioavailability of copper in tribasic copper chloride to copper in copper sulfate for laying hens based on egg yolk and feather copper concentrations. Poult Sci.

[23] Kim JH, Park GH, Han GP, Kil DY (2021). Effect of feeding corn distillers dried grains with solubles naturally contaminated with deoxynivalenol on growth performance, meat quality, intestinal permeability, and utilization of energy and nutrients in broiler chickens. Poult Sci.

[24] Wolynetz MS, Sibbald IR (1984). Relationships between apparent and true metabolizable energy and the effects of a nitrogen correction. Poult Sci.

[25] Cowieson AJ, Bedford MR, Ravindran V (2010). Interactions between xylanase and glucanase in maize-soy-based diets for broilers. Br Poult Sci.

[26] Ebringerová A, Heinze T (2000). Xylan and xylan derivatives–biopolymers with valuable properties, 1. naturally occurring xylans structures, isolation procedures and properties. Macromol Rapid Commun.

[27] Dale T, Hannay I, Bedford MR, Tucker GA, Brameld JM, Parr T (2020). The effects of exogenous xylanase supplementation on the in vivo generation of xylooligosaccharides and monosaccharides in broilers fed a wheat-based diet. Br Poult Sci.

[28] Mandalari G, Mackie AM, Rigby NM, Wickham MSJ, Mills ENC (2009). Physiological phosphatidylcholine protects bovine β-lactoglobulin from simulated gastrointestinal proteolysis. Mol Nutr Food Res.

[29] Greiner R (2021). Limitations of an in vitro model of the poultry digestive tract on the evaluation of the catalytic performance of phytases. J Sci Food Agric.

[30] Suman SP, Joseph P (2013). Myoglobin chemistry and meat color. Annu Rev Food Sci Technol.

[31] Wei Y, Qin K, Qin X, Song F, Xu X (2023). Effects of different types of xanthophyll extracted from marigold on pigmentation of yellow-feathered chickens. Anim Biosci.

[32] Lee C, Kim JH, Kil DY (2022). Comparison of stress biomarkers in laying hens raised under a long-term multiple stress condition. Poult Sci.

[33] Ghasemi HA, Ghasemi R, Torki M (2014). Periodic usage of low-protein methionine-fortified diets in broiler chickens under high ambient temperature conditions: Effects on performance, slaughter traits, leukocyte profiles and antibody response. Int J Biometeorol.

[34] Fatemi M, Toghyani M (2018). Effect of tryptophan supplementation in protein deficient diets on performance, gut development and immune responses in broiler chickens. Iran J Appl Anim Sci.

[35] Nari N, Ghasemi HA, Hajkhodadadi I, Farahani AK (2020). Intestinal microbial ecology, immune response, stress indicators, and gut morphology of male broiler chickens fed low-phosphorus diets supplemented with phytase, butyric acid, or saccharomyces boulardii. Livest Sci.

[36] CVB (2021). CVB Feed Table 2021: Chemical composition and nutritional values of feedstuffs.

[37] Englyst H, Wiggins HS, Cummings JH (1982). Determination of the non-starch polysaccharides in plant foods by Gas-Liquid chromatography of constituent sugars as alditol acetates. Analyst.

